# Supplementary material for: Temporal dynamics of macrophage transcriptional profiles during zebrafish wound healing
Source: Front Immunol. 2026 Jan 19;16:1721612. doi: 10.3389/fimmu.2025.1721612 (PMC12861901; doi:10.3389/fimmu.2025.1721612)
Supplement: Supplementary file 1 [file DataSheet1.pdf]

Supplementary data

Temporal dynamics of macrophage transcriptional profiles during zebrafish wound healing

Authors

Christina Begon-Pescia[1], Laurent Manchon[4], Resul Özbilgiç[1], Stéphanie Boireau[2], Anaïs Louis[3], Simon Georges[3], Mai Nguyen-Chi[1]

Supplementary figure legends:

Figure S1. Statistical analysis of Real Time quantitative PCR kinetic assays.

Bar graphs show the fold change in mRNA expression levels of selected genes (*il-1b*, *il-8*, *tnfa.b* and *nos2a*), as measured by RT-qPCR. Expression was analyzed in whole larvae following caudal fin amputation (CUT condition) compared to uninjured controls (UNcut condition). All values were normalized to the housekeeping gene *ef1a* and calculated using the 2^(-ΔΔCp) method. Each dot represents an individual measurement, and bars indicate the mean of four biological replicates (n = 5) per time point and condition (UNcut / CUT). Statistical significance was assessed using the unpaired, non-parametric Mann-Whitney U test. Significance levels are indicated as $^{*}p < 0.05$, $^{**}p < 0.01$ and $^{***}p < 0.001$ and not significant (ns). Error bars represent standard error of the mean (SEM).

Figure S2. Representative caudal fin images of mCherry-expressing macrophages.

Caudal fin folds were imaged using spinning disk microscope. Images show transmitted light (brightfield), maximum intensity projections of mCherry-F fluorescence (macrophages), and overlays of both channels in non-amputated (UNcut control) and amputated (CUT) *Tg(mfap4:mCherry-F)* zebrafish larvae at 2, 5, and 29 hours post-amputation (hpA). White dotted lines indicate the fin margins and the initial amputation site. Scale bars = 100 µm.

Figure S3. Gating strategy for sorting *mfap4:mCherry-F⁺* cells in a representative FACS experiment.

(A–D) Flow cytometry gating strategy used to isolate *mfap4:mCherry-F⁺* macrophages from whole *Tg(mfap4:mCherry-F)* zebrafish larvae using Fluorescence-Activated Cell Sorting (FACS). **(A)** Initial gating based on Forward SCatter Area (FSC-A) and Side SCatter Area (SSC-A) was used to exclude debris and dead cells by selecting cells based on size and granularity. **(B, C)** Doublet discrimination was performed using SSC-Width (SSC-W) vs SSC-Height (SSC-H) and FSC-Width (FSC-W) vs FSC-Height (FSC-H), allowing the isolation of singlet populations (Singlets 1 and 2, respectively). **(D)** Live cells were distinguished from dead cells using SYTOX™ Red staining, visualized in an FSC-A vs SYTOX™Red dot plot. **(E)** Unlabeled wild-type (WT) cells were used as a negative control. **(G)** FACS profile of *mfap4:mCherry-F⁺* cells from uninjured

(UNcut) larvae. **(I)** FACS profile of *mfap4:mCherry-F⁺* cells from injured (CUT) larvae. To ensure high purity, gating was optimized to avoid population overlap. **(F, H, J)** Summary tables show the gating statistics, including total number of events (# Events), percentage of the parent population (% Parent), and percentage of the total population (% Total). The percentage of *mfap4:mCherry-F⁺* cells is highlighted in red boxes. Abbreviations: FSC-A = forward scatter area; SSC-A = side scatter area; FSC-H = forward scatter height; FSC-W = forward scatter width; SSC-H = side scatter height; SSC-W = side scatter width.

Figure S4. Expression of Macrophage-Specific Marker Genes in Sorted Populations.

MCherry-F⁺ cells were isolated by FACS from caudal fin-amputated larvae at 2, 5, and 29 hours post-amputation (hpA). Normalized expression of several key macrophage markers highly enriched in mCherry-F⁺ cells. Bar colors indicate the respective time points. Gene expression levels are shown as normalized raw counts obtained by Illumina RNA sequencing. The expression profiles confirm the identity of the macrophage population across all sampled time points.

Figure S5. Quantitative real-time PCR (RT-qPCR) validation of RNA-sequencing results. Bar graphs show the relative mRNA expression levels of selected differentially expressed genes (DEGs), as measured by RT-qPCR. Expression levels were analyzed in FACS-sorted macrophages from larvae with caudal fin amputation (CUT condition) compared to macrophages from uninjured larvae (UNcut control condition). All values were normalized to the *ef1a* housekeeping gene and calculated using the $2^{(-\Delta Ct)}$ method. Each dot represents a measured value and bars represent mean from 3 to 7 biological replicates per time point. Statistical significance was assessed using the unpaired, non-parametric Mann-Whitney U test. Significance levels are indicated as $^{*}p < 0.05$, $^{**}\ p < 0.01$ and $^{***}\ p < 0.001$ and not significant (ns). Error bars represent standard error of the mean (SEM). **(A, B)** Down- and up-regulated genes at 2 hpA. **(C, D)** Down- and up-regulated genes at 5 hpA. **(E, F)** Down- and up-regulated genes at 29 hpA.

Figure S6. Enlarged version of Figure 3A

Heat-map of differentially expressed genes. The 50 most strongly up- and down-regulated genes are displayed. For each gene, normalized read counts were Z-score–transformed across all samples (columns = individual biological replicates). Colour scale: dark brown = high expression; light yellow = low expression. Clusters enriched in up-regulated genes are marked with red outlines; clusters enriched in down-regulated genes are marked in blue. Samples from amputated larvae (CUT) are labelled with green squares, and control samples (UNcut) with purple squares. Hierarchical clustering was performed with Ward's minimum-variance method on $\log_2$ fold-change values.

Figure S7. Enlarged version of Figure 3D

GO chord plot of Kyoto Encyclopedia of Genes and Genomes (KEGG)-associated terms. Genes are connected to their associated pathways by ribbons. Genes are ordered by $\log_2$ fold change (red = strong up-regulation; blue = strong down-regulation).

Figure S8. Gene Ontology enrichment analysis on shared genes between early wound and early *Salmonella* infection.

Bubble plots of Gene Ontology enrichment. Biological-process (**A**), molecular-function (**B**) terms and (KEGG)-associated terms (**C**) enriched among the up-regulated DEGs that are shared between early wound and early *Salmonella* infection. Dot size represents the number of genes in each term; dot colour encodes significance (FDR): red = higher significance (lower *p*-value), green = lower significance (higher *p*-value). The x-axis shows fold enrichment (gene ratio); the y-axis lists enriched GO terms.

Figure S9: comparison of *Tg(mfap4:mCherry-F)* and *Tg(mpx:GFP)* lines.

(A) Representative images of the whole *Tg(mfap4:mCherry-F/mpx:GFP)* larvae at 60 hpf acquired using an epi-fluorescence microscope. Merge images and fluorescence of mCherry-F (macrophages) and GFP (neutrophils) are shown. Scale bar: 100 μM. **(B)** The caudal fin fold of *Tg(mfap4:mCherry-F/mpx:GFP)* larvae was amputated at 3 dpf. Representative images of the fin fold at 6, 9 and 11 hpA. Images are maximum projections from confocal microscopy imaging showing the fluorescence of mCherry-F (macrophages) and GFP (neutrophils) in the fin folds. Scale bar: 50 μM

List of tables:

- Table 1. RT-qPCR Primer details for genes validation RNA-Sequencing findings.
- Table 2. List of DEGs (CUT vs UNcut) at 2hpA obtained using DESeq2 analysis
- Table 3. List of DEGs (CUT vs UNcut) at 5hpA obtained using DESeq2 analysis
- Table 4. List of DEGs (CUT vs UNcut) at 29hpA obtained using DESeq2 analysis
- Table 5. List of the top DEGs (CUT vs UNcut) at 2hpA
- Table 6. Gene ontology analysis at 2hpA in the category Molecular Function
- Table 7. KEGG Pathway enrichment analysis at 2hpA
- Table 8. Commonly up- and down- DEGs in wound and infection condition

- Table 9. List of the top DEGs (CUT vs UNcut) at 5hpA
- Table 10. KEGG Pathway enrichment analysis at 5hpA
- Table 11. Gene ontology analysis at 5hpA in the category Molecular Function
- Table 12. Gene ontology analysis at 5hpA in the category Biological Process
- Table 13. List of Genes shared between 2hpA, 5hpA and 29hpA

Figure S1

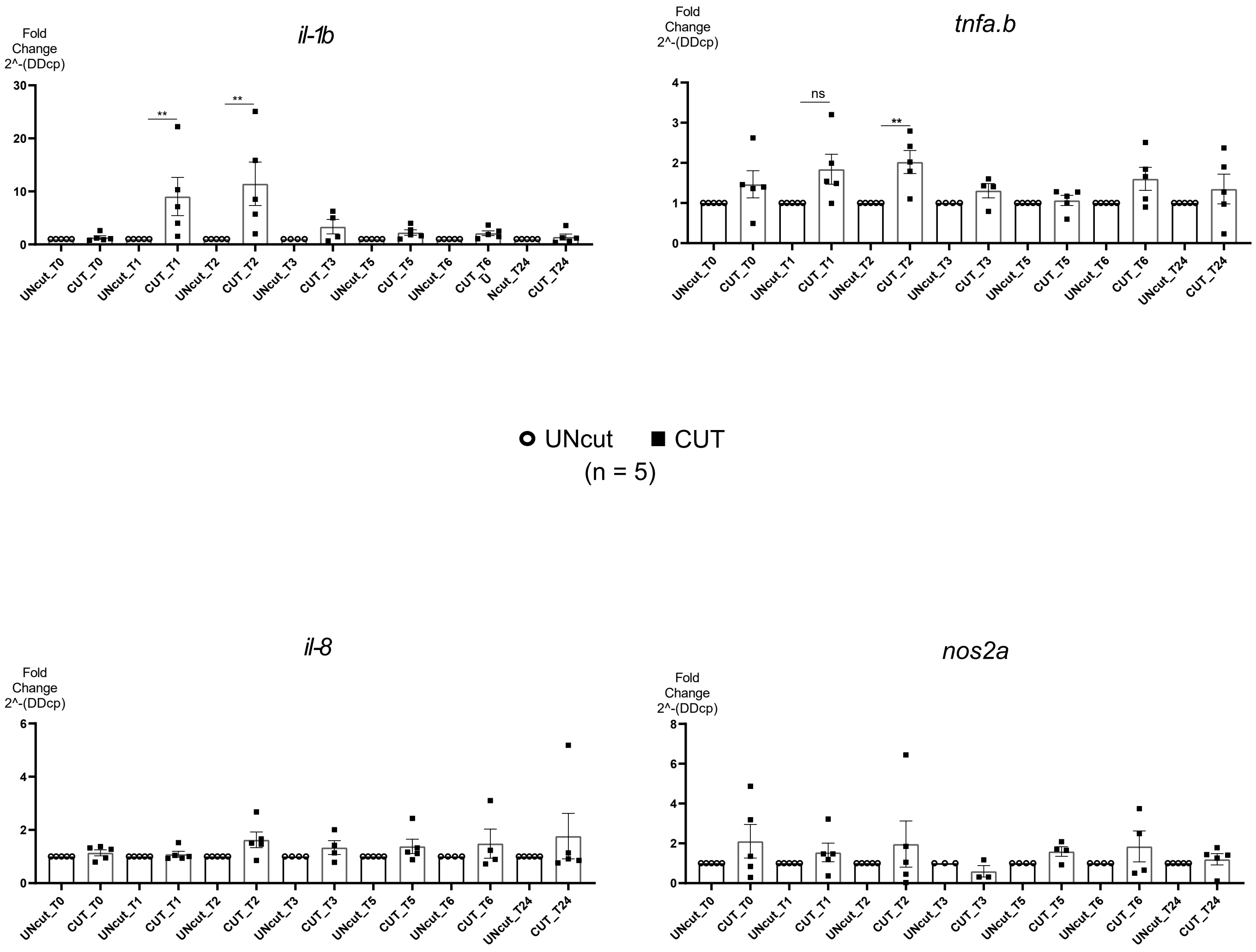

il-1b
Fold Change 2^-(DDcp)
30
20
10
0
**
**
UNcut_T0
CUT_T0
UNcut_T1
CUT_T1
UNcut_T2
CUT_T2
UNcut_T3
CUT_T3
UNcut_T5
CUT_T5
UNcut_T6
CUT_T6
UNcut_T24
CUT_T24
tnfa.b
Fold Change 2^-(DDcp)
4
3
2
1
0
ns
**
UNcut_T0
CUT_T0
UNcut_T1
CUT_T1
UNcut_T2
CUT_T2
UNcut_T3
CUT_T3
UNcut_T5
CUT_T5
UNcut_T6
CUT_T6
UNcut_T24
CUT_T24
UNcut
CUT
(n = 5)
il-8
Fold Change 2^-(DDcp)
6
4
2
0
UNcut_T0
CUT_T0
UNcut_T1
CUT_T1
UNcut_T2
CUT_T2
UNcut_T3
CUT_T3
UNcut_T5
CUT_T5
UNcut_T6
CUT_T6
UNcut_T24
CUT_T24
nos2a
Fold Change 2^-(DDcp)
8
6
4
2
0
UNcut_T0
CUT_T0
UNcut_T1
CUT_T1
UNcut_T2
CUT_T2
UNcut_T3
CUT_T3
UNcut_T5
CUT_T5
UNcut_T6
CUT_T6
UNcut_T24
CUT_T24

Figure S2

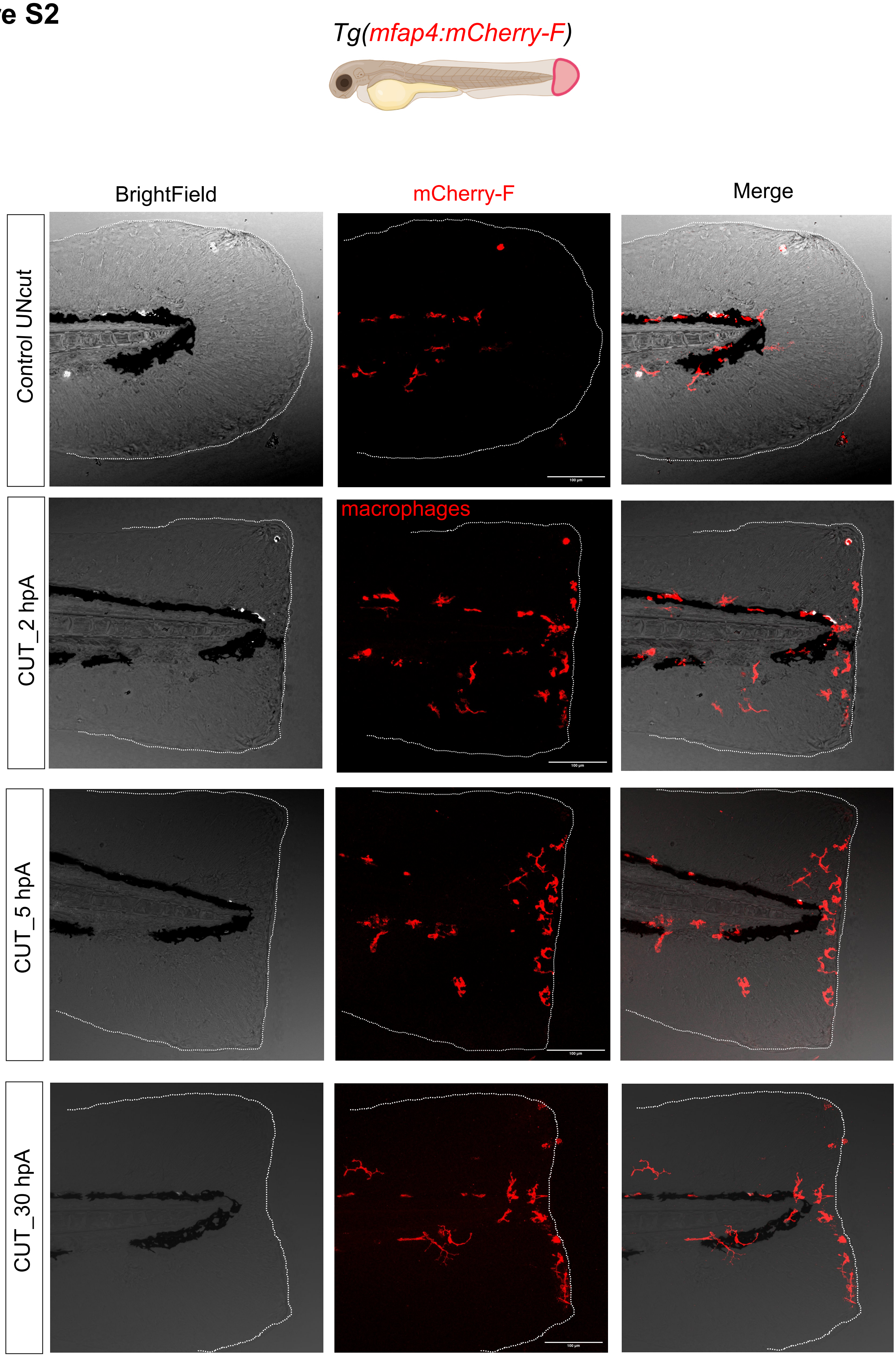
Tg(mfap4:mCherry-F)
BrightField
mCherry-F
Merge
Control UNcut
CUT_2 hpA
CUT_5 hpA
CUT_30 hpA
macrophages

Figure S3

A

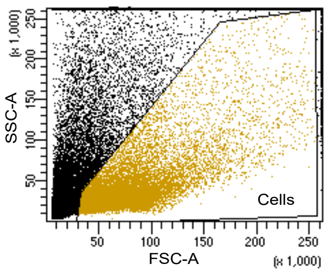

B

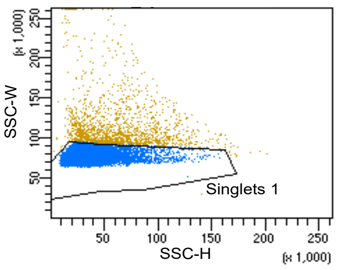

C

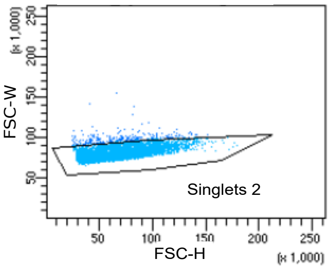

D

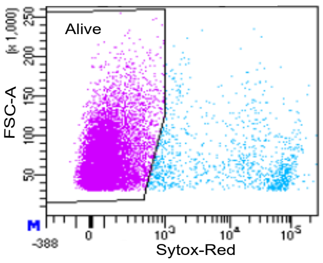

E

Unlabeled cells

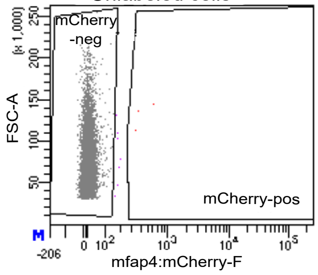

F

Unlabeled cells

Population	#Events	%Parent	%Total
All Events	40,000	####	100.0
Cells	16,828	42.1	42.1
Singlets 1	15,229	90.5	38.1
Singlets 2	15,003	98.5	37.5
Alive	13,847	92.3	34.6
mCherry-pos	3	0.0	0.0
mCherry-neg	13,837	99.9	34.6

G

UNcut_*mfap4:mCherry-F*

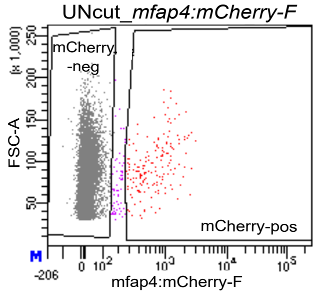

H

UNcut_*mfap4:mCherry-F*

Population	#Events	%Parent	%Total
All Events	40,000	####	100.0
Cells	17,396	43.5	43.5
Singlets 1	14,258	82.0	35.6
Singlets 2	13,857	97.2	34.6
Alive	12,980	93.7	32.4
mCherry-pos	172	1.3	0.4
mCherry-neg	12,743	98.2	31.9

I

CUT_*mfap4:mCherry-F*

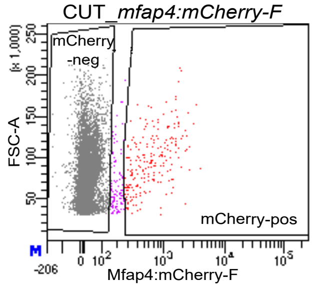

J

CUT_*mfap4:mCherry-F*

Population	#Events	%Parent	%Total
All Events	40,000	####	100.0
Cells	16,814	42.0	42.0
Singlets 1	14,786	87.9	37.0
Singlets 2	14,553	98.4	36.4
Alive	13,551	93.1	33.9
mCherry-pos	223	1.6	0.6
mCherry-neg	13,212	97.5	33.0

Figure S4

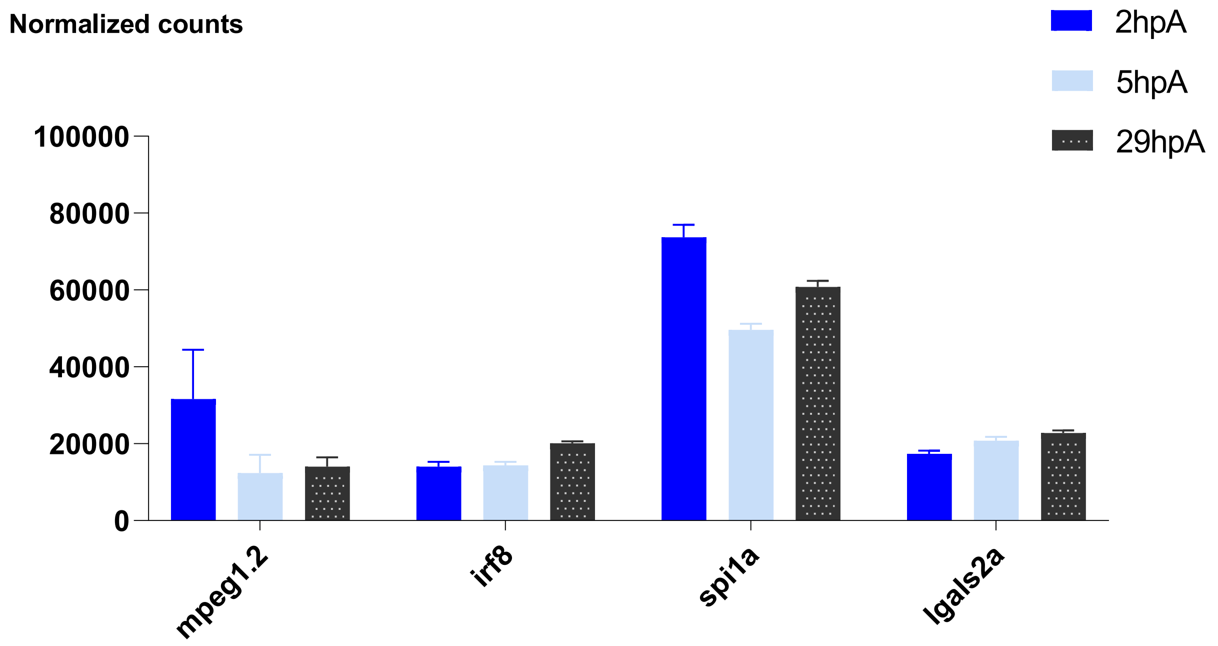
Normalized counts
100000
80000
60000
40000
20000
0
mpeg1.2
irf8
spi1a
lgals2a
2hpA
5hpA
29hpA

Figure S5

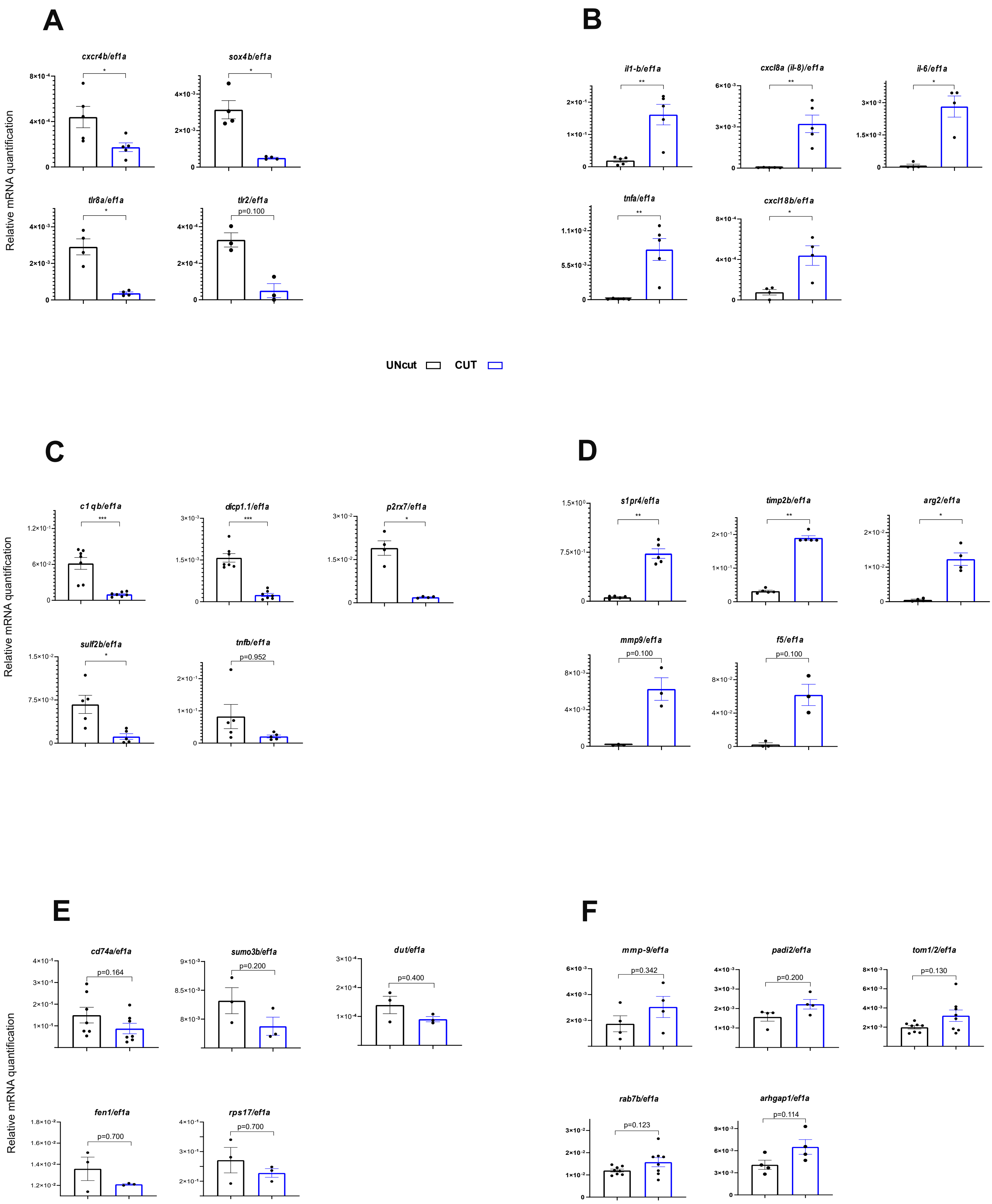
A
B
C
D
E
F
Relative mRNA quantification
cxcr4b/ef1a
sox4b/ef1a
tlr8a/ef1a
tlr2/ef1a
p=0.100
il1-b/ef1a
cxcl8a (il-8)/ef1a
il-6/ef1a
tnfa/ef1a
cxcl18b/ef1a
UNcut
CUT
c1qb/ef1a
dicp1.1/ef1a
p2rx7/ef1a
sulf2b/ef1a
tnfb/ef1a
p=0.952
s1pr4/ef1a
timp2b/ef1a
arg2/ef1a
mmp9/ef1a
p=0.100
f5/ef1a
p=0.100
cd74a/ef1a
p=0.164
sumo3b/ef1a
p=0.200
dut/ef1a
p=0.400
fen1/ef1a
p=0.700
rps17/ef1a
p=0.700
mmp-9/ef1a
p=0.342
padi2/ef1a
p=0.200
tom1/2/ef1a
p=0.130
rab7b/ef1a
p=0.123
arhgap1/ef1a
p=0.114

Figure S6

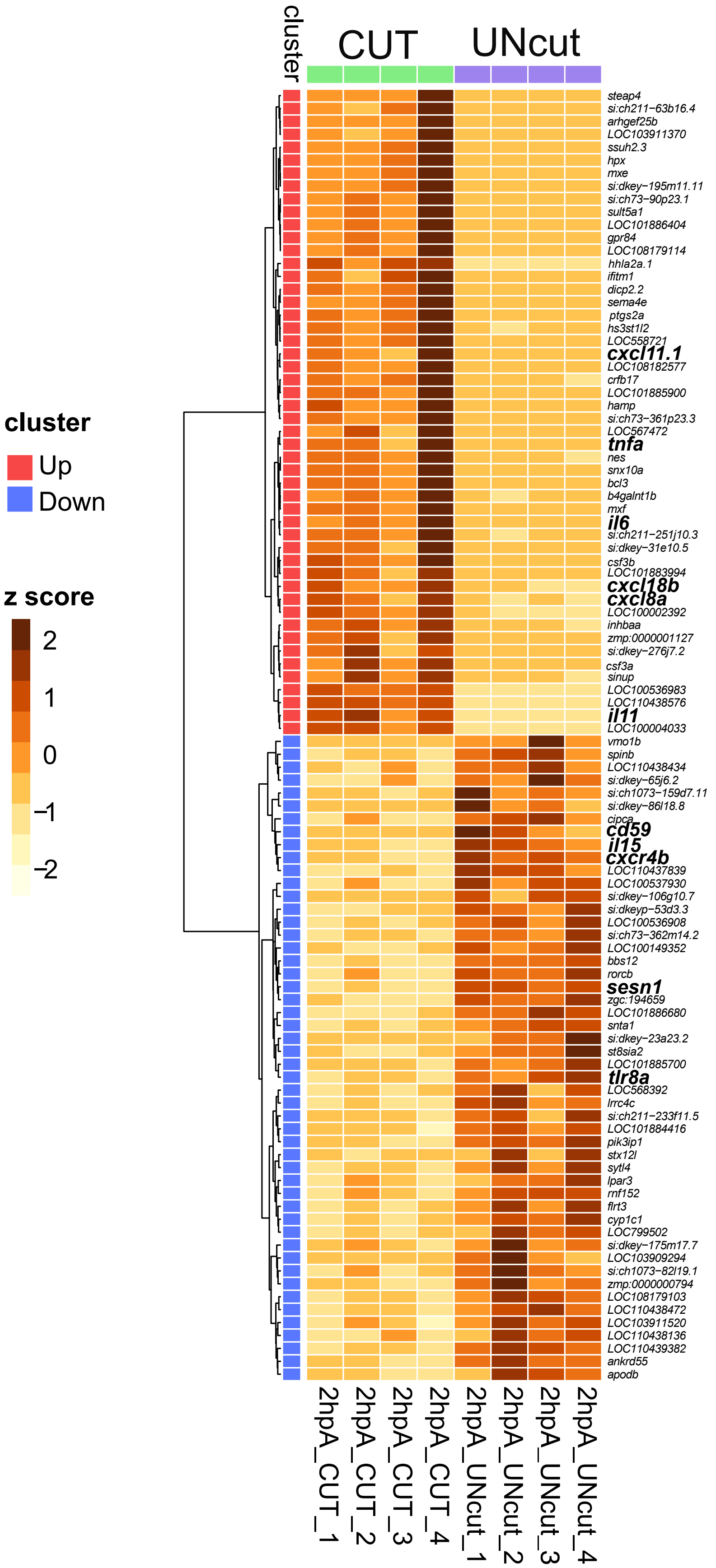
cluster
CUT
UNcut
cluster
Up
Down
z score
2
1
0
−1
−2
steap4
si:ch211−63b16.4
arhgef25b
LOC103911370
ssuh2.3
hpx
mxe
si:dkey−195m11.11
si:ch73−90p23.1
sult5a1
LOC101886404
gpr84
LOC108179114
hhla2a.1
ifitm1
dicp2.2
sema4e
ptgs2a
hs3st1l2
LOC558721
cxcl11.1
LOC108182577
crfb17
LOC101885900
hamp
si:ch73−361p23.3
LOC567472
tnfa
nes
snx10a
bcl3
b4galnt1b
mxf
il6
si:ch211−251j10.3
si:dkey−31e10.5
csf3b
LOC101883994
cxcl18b
cxcl8a
LOC100002392
inhbaa
zmp:0000001127
si:dkey−276j7.2
csf3a
sinup
LOC100536983
LOC110438576
il11
LOC100004033
vmo1b
spinb
LOC110438434
si:dkey−65j6.2
si:ch1073−159d7.11
si:dkey−86l18.8
cipca
cd59
il15
cxcr4b
LOC110437839
LOC100537930
si:dkey−106g10.7
si:dkeyp−53d3.3
LOC100536908
si:ch73−362m14.2
LOC100149352
bbs12
rorcb
sesn1
zgc:194659
LOC101886680
snta1
si:dkey−23a23.2
st8sia2
LOC101885700
tlr8a
LOC568392
lrrc4c
si:ch211−233f11.5
LOC101884416
pik3ip1
stx12l
sytl4
lpar3
rnf152
flrt3
cyp1c1
LOC799502
si:dkey−175m17.7
LOC103909294
si:ch1073−82l19.1
zmp:0000000794
LOC108179103
LOC110438472
LOC103911520
LOC110438136
LOC110439382
ankrd55
apodb
2hpA_CUT_1
2hpA_CUT_2
2hpA_CUT_3
2hpA_CUT_4
2hpA_UNcut_1
2hpA_UNcut_2
2hpA_UNcut_3
2hpA_UNcut_4

Figure S7

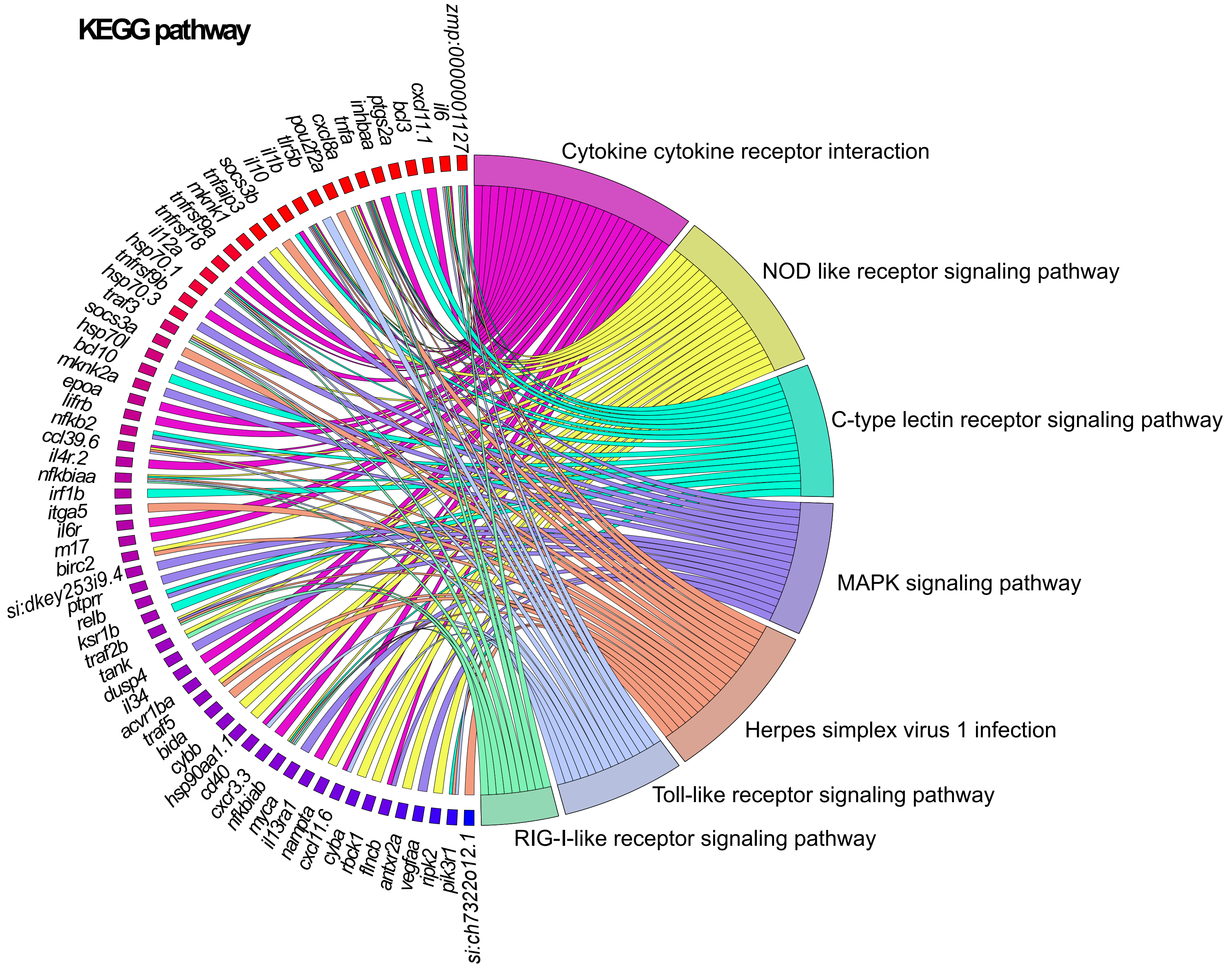
KEGG pathway
Cytokine cytokine receptor interaction
NOD like receptor signaling pathway
C-type lectin receptor signaling pathway
MAPK signaling pathway
Herpes simplex virus 1 infection
Toll-like receptor signaling pathway
RIG-I-like receptor signaling pathway
zmp:0000001127
il6
cxcl11.1
bcl3
ptgs2a
inhbaa
tnfa
cxcl8a
pou2f2a
tlr5b
il1b
il10
socs3b
tnfaip3
mknk1
tnfrsf9a
tnfrsf18
il12a
hsp70.1
tnfrsf9b
hsp70.3
traf3
socs3a
hsp70l
bcl10
mknk2a
epoa
lifrb
nfkb2
ccl39.6
il4r.2
nfkbiaa
irf1b
itga5
il6r
m17
birc2
si:dkey253i9.4
ptprr
relb
ksr1b
traf2b
tank
dusp4
il34
acvr1ba
traf5
bida
cybb
hsp90aa1.1
cd40
cxcr3.3
nfkbiab
myca
il13ra1
nampta
cxcl11.6
cyba
rbck1
flncb
antxr2a
vegfaa
ripk2
pik3r1
si:ch73220o12.1
logFC
1.5
2.0
2.5
3.0

Figure S8

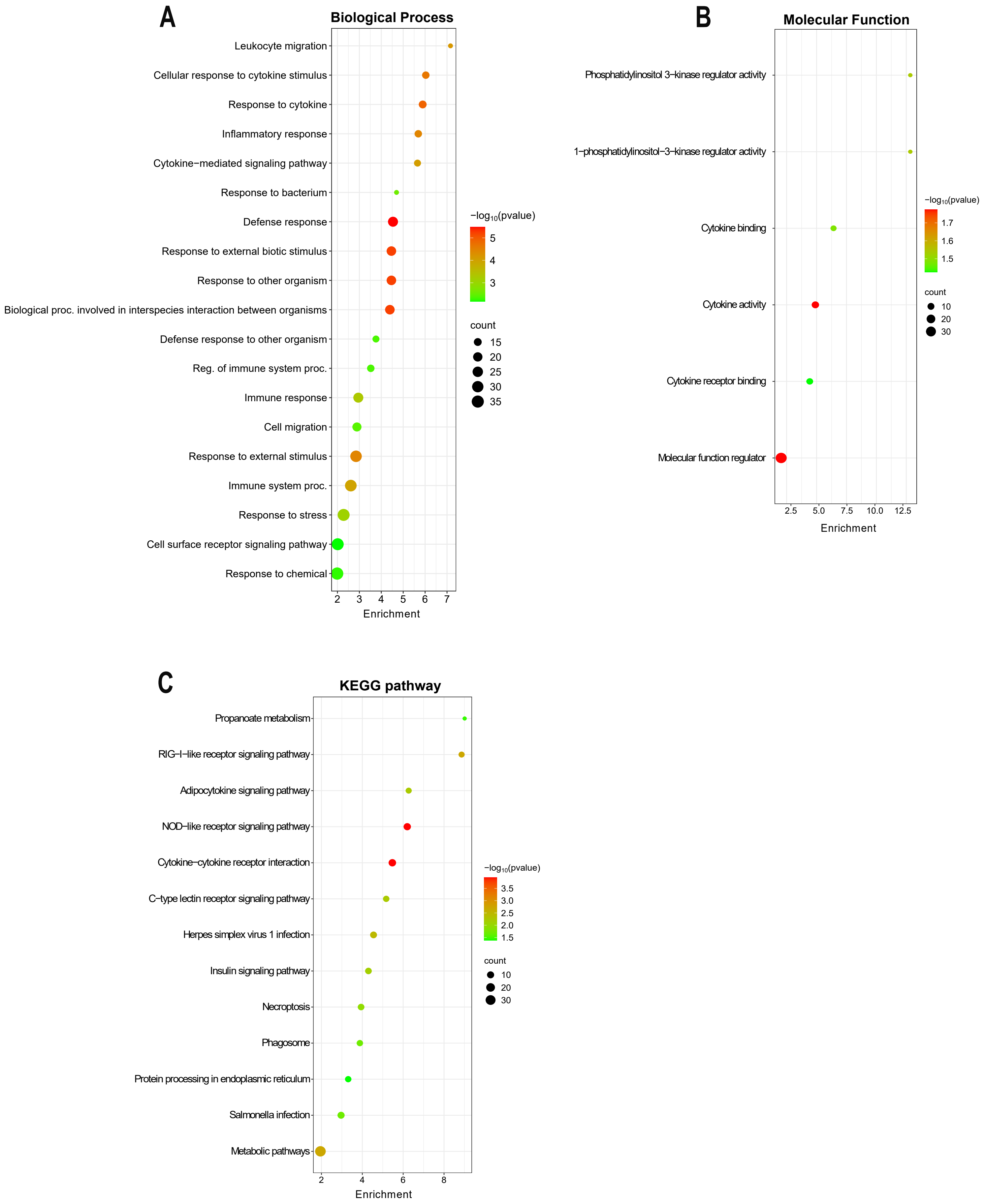

A
Biological Process
Leukocyte migration
Cellular response to cytokine stimulus
Response to cytokine
Inflammatory response
Cytokine−mediated signaling pathway
Response to bacterium
Defense response
Response to external biotic stimulus
Response to other organism
Biological proc. involved in interspecies interaction between organisms
Defense response to other organism
Reg. of immune system proc.
Immune response
Cell migration
Response to external stimulus
Immune system proc.
Response to stress
Cell surface receptor signaling pathway
Response to chemical
2 3 4 5 6 7
Enrichment
−log10(pvalue)
5
4
3
count
15
20
25
30
35
B
Molecular Function
Phosphatidylinositol 3−kinase regulator activity
1−phosphatidylinositol−3−kinase regulator activity
Cytokine binding
Cytokine activity
Cytokine receptor binding
Molecular function regulator
2.5 5.0 7.5 10.0 12.5
Enrichment
−log10(pvalue)
1.7
1.6
1.5
count
10
20
30
C
KEGG pathway
Propanoate metabolism
RIG−I−like receptor signaling pathway
Adipocytokine signaling pathway
NOD−like receptor signaling pathway
Cytokine−cytokine receptor interaction
C−type lectin receptor signaling pathway
Herpes simplex virus 1 infection
Insulin signaling pathway
Necroptosis
Phagosome
Protein processing in endoplasmic reticulum
Salmonella infection
Metabolic pathways
2 4 6 8
Enrichment
−log10(pvalue)
3.5
3.0
2.5
2.0
1.5
count
10
20
30

Figure S9

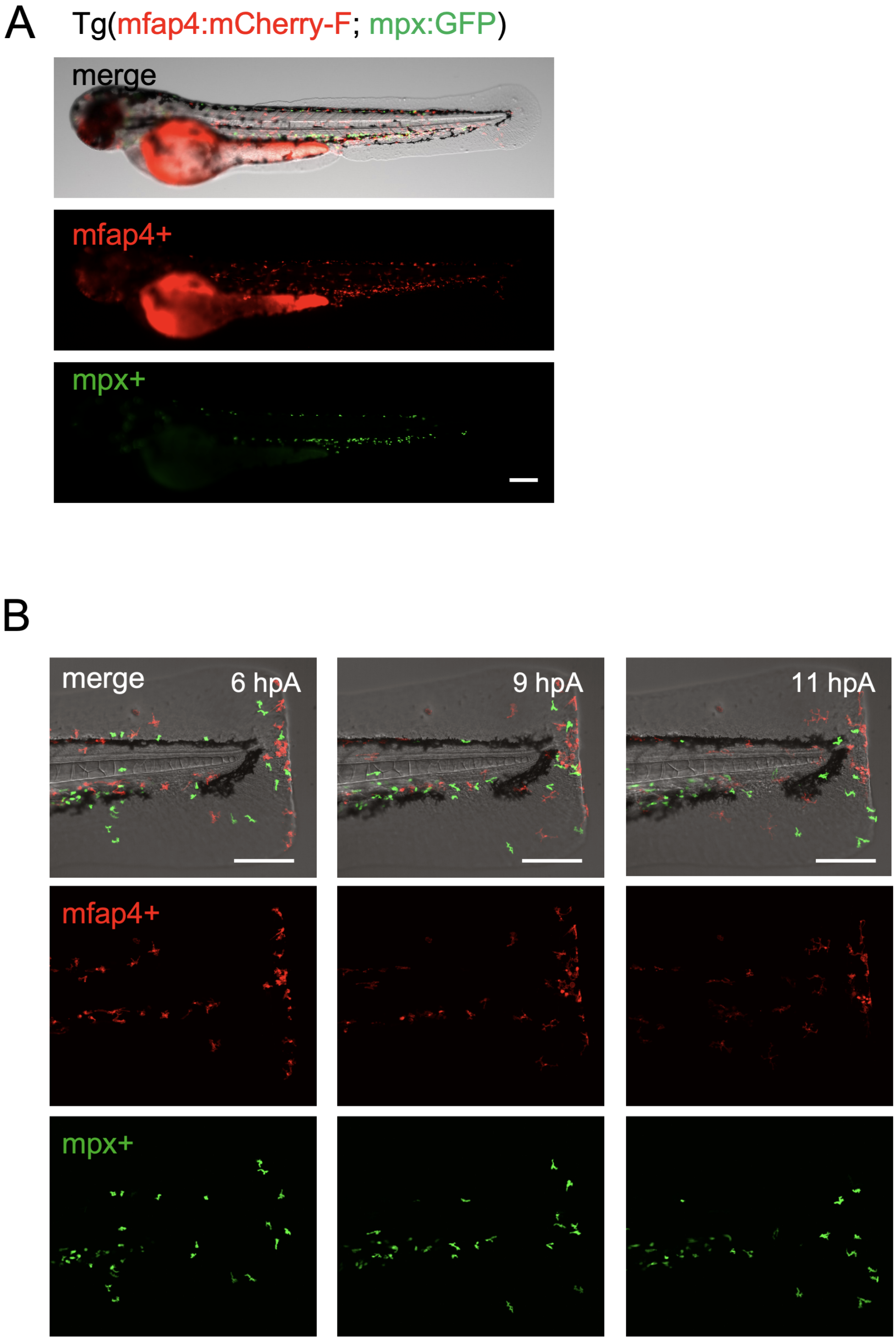
A
Tg(mfap4:mCherry-F; mpx:GFP)
merge
mfap4+
mpx+
B
merge
6 hpA
9 hpA
11 hpA
mfap4+
mpx+
